# Attitudes Towards the Use of Artificial Intelligence in Dermatology: A Survey of Australian Dermatologists

**DOI:** 10.1111/ajd.14524

**Published:** 2025-05-14

**Authors:** Brad Partridge, Monika Janda, Nicole Gillespie, Carina Vasconcelos Silva, Chris Arnold, Lisa Abbott, Tony Caccetta, H. Peter Soyer

**Affiliations:** ^1^ Centre for Health Services Research The University of Queensland Brisbane Queensland Australia; ^2^ Melbourne Business School University of Melbourne Melbourne Victoria Australia; ^3^ School of Business The University of Queensland Brisbane Queensland Australia; ^4^ Hodgson Associates BioGrid Australia Ltd Parkville Victoria Australia; ^5^ Hodgson Associates & Biogrid Australia Ltd, Parkville, Victoria Australia; ^6^ Melanoma Institute of Australia & University of New South Wales, Sydney, New South Wales Australia; ^7^ School of Medicine The University of Western Australia Perth Western Australia Australia; ^8^ Frazer Institute, The University of Queensland, Dermatology Research Centre Brisbane Queensland Australia

**Keywords:** artificial intelligence, dermatology, healthcare technology acceptance, skin cancer, survey research, trust

## Abstract

**Background/Objectives:**

This study explored the views of dermatologists in Australia on the use of Artificial Intelligence (AI) in dermatology.

**Method:**

Fellows and Trainees of the Australasian College of Dermatologists (ACD) were invited to participate in an anonymous online survey, resulting in a sample of 122 completed surveys (response rate 16.2%).

**Results:**

Although 44% have used AI in their dermatology practice, only a minority are using AI regularly for clinical (12%) or administrative purposes (17%). A key barrier is trust, with most (69%) participants either unwilling or unsure about trusting AI for supporting the diagnosis of skin cancer. Participants identified accuracy and information on datasets, limitations, benefits, and the purpose of AI as important for trust. 52% want AI accuracy to be equivalent to or superior to the best dermatologist if it is used for clinical diagnosis. Participants perceived a range of benefits and risks of AI, with key risks around accuracy and divestment of AI to tech companies, and key benefits being reduction of monotonous tasks and improved patient access. Few (10%) are concerned about AI replacing dermatologists even though almost half (47%) expect key aspects of dermatology work will be performed by AI.

**Conclusions:**

Dermatologists in Australia are at an early stage of integrating AI into practice, with most wary or unsure about the accuracy of currently available AI tools for diagnostic purposes. Developing workflows that are acceptable to clinicians may require knowledge outreach from Dermatology Colleges, such as the ACD, to help clinicians develop well informed views on AI.

## 1 Introduction

The use of Artificial Intelligence (AI) has the potential to significantly impact dermatology, particularly in the detection and management of skin cancer. AI tools have been shown to correctly identify images of malignant skin lesions under experimental conditions with a level of diagnostic accuracy comparable to experts [1, 2, 3]. Integrating AI with 2D or 3D skin imaging technologies into clinical workflows is seen as a promising way of improving access to accurate, cost‐effective, screening and diagnosis [4, 5]. In countries with a high prevalence of skin cancer and high consumer demand for dermatological care, such as Australia, this may lead to considerably better outcomes for patients and savings to the healthcare system [5]. Given the increasing interest in AI, the Australasian College of Dermatologists (ACD) issued a 2022 position statement that “encourages and supports ongoing efforts to develop AI for dermatology” with guidelines intended to foster AI's safe, ethical, and effective clinical use [6].

Although demonstrating the technical capabilities of AI in experimental settings is important, the integration of AI into practice will also be influenced by dermatologists' understanding of AI. Their views on the potential benefits and drawbacks of AI for their clinical decision making, their level of trust in the technology, and whether they see AI as a benefit to patients will all bear upon decisions to incorporate AI. It is therefore important to understand the attitudes of dermatologists towards the use of AI, as well as their expectations for its clinical use, and whether and how they are currently incorporating AI tools into their practice.

Only a small number of international surveys have explored dermatologists' views on AI use (including Europe, China, the Middle East, and the USA) [7, 8, 9, 10, 11, 12, 13]. Despite few dermatologists feeling they have a good knowledge or understanding of AI [9, 12], these surveys show that there is a general expectation among dermatologists that AI‐supported decision making will have a positive impact on the field [8, 11, 13, 14] and many are amenable to AI helping inform their diagnostic or management decisions [10, 11]. Potential clinical workflows whereby clinicians review diagnostic decisions made by AI have mostly been met with support [11, 14]; however, dermatologists also frequently report concerns. These include: insufficient accuracy for use in clinical settings [14]; the de‐skilling of clinicians [11]; AI being “over trusted” by clinicians [10]; and data privacy issues [10]. Nevertheless, the extent to which these views easily generalise across disparate healthcare systems and cultural contexts is unclear, meaning that locally relevant surveys are needed.

To date, a 2021 survey with radiologists, ophthalmologists, radiation oncologists, and dermatologists [14] is the only published study of views towards AI among dermatologists working in Australia. Among the dermatologists in that study (*n* = 97), the most commonly anticipated benefits of AI were the potential for it to improve patient access to disease screening and improve diagnostic confidence. However, concerns about how to manage AI error (and any potential medicolegal risks) were common, and 63% were of the view that AI tools need to be at least as good as the best performing dermatologist in order to be used for the purpose of diagnostic decision‐support. When that survey data was collected (in 2019), 46% of dermatologists expected AI to have a noticeable impact on the field within the proceeding 5 years (i.e., by 2024) [14]. Since 2019, AI investment and development has increased markedly, and a number of commonly used skin imaging platforms have already incorporated AI functions that allow clinicians to use these features (noting they are yet to be granted formal regulatory approval as diagnostic tools in Australia).

To ensure developers, regulators, and policy makers are appropriately informed of and responsive to the views of clinicians, the aim of this study is to generate an up‐to‐date snapshot of attitudes among dermatologists in Australia towards the use of AI tools (either for clinical purposes or for administrative tasks).

## 2 Method

### 2.1 Survey

An online survey using the Qualtrics platform was developed by the research team. The survey consisted of 7 demographic questions and 12 survey questions to assess current knowledge and use of AI in dermatology; trust in AI; potential benefits and risks related to the use of AI; attitudes towards a potential clinical workflow for incorporating AI into a widespread melanoma screening program; and views on the role of the ACD. Survey items used Likert scales and some open‐ended response boxes to enable qualitative responses. Items were either constructed by the research team or adapted from the study by Scheetz et al. [14] to allow for direct comparisons.

### 2.2 Sample and Recruitment

Participants were fellows and trainees of the Australasian College of Dermatologists (the specialist medical college that conducts training and continuing professional development required for registration to practice as a dermatologist in Australia). Invitations to participate, including the link to the online survey, were emailed to all ACD fellows and trainees weekly during April 2024 through the ACD newsletter. The survey link was also advertised to attendees of the ACD Annual Conference in May 2024. Participation in the online survey was anonymous and could in no way be linked to participant email addresses or any other identifying information. The final sample included 122 completed surveys (75 Female, 44 Male, 3 Non‐binary), response rate 16.2%. The sample comprised 105 ACD fellows (86.1%) and 17 ACD trainees (13.9%). Average age was 49.9 years (range 29–93), and average years of experience in dermatology was 17.5 years (range 1–50).

## 3 Results

### 3.1 Knowledge of AI and Current Use

Most participants rated their knowledge of AI applications to dermatology as poor or fair (75.4%), with only 24.6% rating their knowledge of AI as good or excellent (see Table 1). Participants were asked about their use of AI for clinical purposes such as identifying new or changing lesions, informing diagnostic decisions, or getting a second opinion. Overall use was low, with 56.6% having never used AI for clinical tasks and a further 31.3% using AI for clinical purposes only rarely. 12.3% of participants reported using AI for clinical purposes sometimes or almost always. Similarly, when asked about their use of AI tools (e.g., ChatGPT) for administrative tasks such as recording patient notes, writing correspondence to patients, or booking appointments, 54.9% of participants reported never having used AI for administrative tasks and a further 25.4% saying only rarely (17.3% said they use AI for administrative tasks sometime to almost always).

**TABLE 1:** Knowledge, use, and attitudes towards AI.

Item	Response	Results *n* (%)
Knowledge of AI and its application in dermatology	Poor	37 (30.3%)
	Fair	55 (45.1%)
	Good	25 (20.5%)
	Excellent	5 (4.1%)
Use AI for clinical purposes	Never	69 (56.6%)
	Rarely	38 (31.1%)
	Sometimes	10 (8.2%)
	Often	0 (0%)
	Almost always	5 (4.1%)
Use AI for administrative tasks	Never	67 (54.9%)
	Rarely	31 (25.4%)
	Sometimes	10 (8.2%)
	Often	3 (2.5%)
	Almost always	8 (6.6%)
	Missing data	3 (2.5%)
Willingness to trust the use of AI tools for skin cancer diagnosis	Very unwilling	18 (14.8%)
	Somewhat unwilling	30 (24.6%)
	Neither willing nor unwilling	19 (15.6%)
	Somewhat willing	26 (21.3%)
	Very willing	4 (3.3%)
	Lack experience to assess	17 (13.9%)
	Missing data	8 (6.6%)
Accuracy of AI tools for supporting clinicians in the diagnosis of skin cancer	Poor	17 (13.9%)
	Fair	37 (30.3%)
	Good	10 (8.2%)
	Excellent	0 (0%)
	Lack experience to assess	50 (41%)
	Missing data	8 (6.6%)
Key aspects of dermatology work will be performed by AI	Strongly disagree	11 (9.0%)
	Somewhat disagree	18 (14.8%)
	Neither agree nor disagree	18 (14.8%)
	Somewhat agree	41 (33.6%)
	Strongly agree	16 (13.1%)
	Unsure	3 (2.5%)
	Missing data	15 (12.3%)
Dermatologists will be replaced by AI	Strongly disagree	42 (34.4%)
	Somewhat disagree	36 (29.5%)
	Neither agree nor disagree	17 (13.9%)
	Somewhat agree	10 (8.2%)
	Strongly agree	2 (1.6%
	Unsure	0 (0.0%)
	Missing data	15 (12.3%)

Almost half (46.7%) thought that key aspects of dermatology work will be performed by AI (23.8% disagreed), but only 9.8% thought that dermatologists will be replaced by AI (63.9% disagreed). See Table 1. Most participants thought their patients were willing to have AI tools incorporated into practice for clinical decisions, diagnosis, and treatment (59.9%), and slightly more for administrative tasks (67.2%).

### 3.2 AI for Skin Cancer Diagnosis—Trust and Accuracy

Only a quarter (24.6%) of participants were willing to trust AI for diagnosing skin cancer, while 39.4% were unwilling to trust AI. A sizeable minority were either unsure or felt unable to make an assessment due to their lack of experience with AI (29.5% combined) (see Table 1). A range of factors were seen by a very high proportion of participants as being essential/highly important to know or understand, to trust the use of AI in dermatology. In particular, the accuracy and performance indicators of the AI tool (91% of the sample rated this as essential/highly important); the limitations of the AI tool (90.2%); information on datasets and populations (82%); the intended purpose of AI (81.2%); and the benefits of AI (77.9%).

44.2% of participants rated the current accuracy of AI tools for supporting the diagnosis of skin cancer as poor/fair (see Table 1). Only 8.2% rated the accuracy of AI as good, but again a large minority of the sample (41%) felt they did not have enough experience with AI to make a good assessment. Given the importance of AI accuracy, we asked participants what level of error would be acceptable for an AI system to be used for diagnostic decision support (see Table 2). The expected standard was high, with 30.3% saying that for AI to be used, its error rate must match the best performing dermatologist (comparable to the 32% reported in the 2021 survey [14]). A further 22.1% are of the view that AI must have a lower error rate than the best performing dermatologist (slightly lower than the 30.9% in the 2021 survey [14]).

**TABLE 2:** What level of error is acceptable for AI when used for the following purposes?

	Superior to the best	Equivalent to the best	Superior to the average	Equivalent to the average	Equivalent to the worst	Missing
Melanoma screening by non‐specialist healthcare workers	18 (14.8%)	28 (23%)	29 (23.8%)	30 (24.6%)	9 (7.4%)	8 (6.6%)
Diagnostic decision‐support for dermatologist	27 (22.1%)	37 (30.3%)	22 (18%)	23 (18.9%)	5 (4.1%)	8 (6.6%)

### 3.3 Benefits and Risks of AI

**FIGURE 1:**
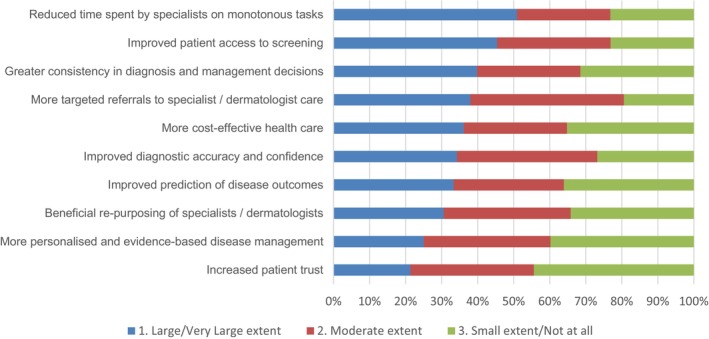
To what extent do you expect these potential benefits from the use of AI in dermatology.

**FIGURE 2:**
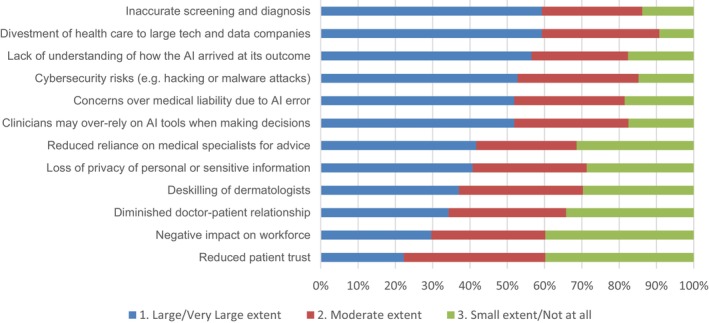
How concerned are you about these potential risks of AI use in dermatology?

We asked participants about the extent to which they expect AI to result in a range of potential benefits in dermatology (see Figure 1). The four biggest expected benefits (large/very large extent) were “reduced time spent by specialists on monotonous tasks” (50.9% of participants); “improved patient access to screening” (45.4%); “greater consistency in diagnosis and management decisions” (39.8%); “more targeted referrals to specialist care” (38.0%). In parallel, we also assessed how concerned participants are about a range of potential risks from the use of AI in dermatology (see Figure 2). The four most strongly held concerns (i.e., large/very large extent) about using AI were the “risk of inaccurate screening or diagnosis” (59.3%); “divestment of health care to large technology and data companies” (59.3%); “lack of understanding of how the AI arrived at its outcome” (56.5%), and “cybersecurity risks (e.g., hacking or malware attacks)” (52.8%).

### 3.4 A Potential Clinical Workflow Using AI for Melanoma Screening

AI may be incorporated into clinical workflows within dermatology in a wide range of ways, and at different points in the decision‐making process. We explored views on a potential clinical workflow that proposed using AI to identify suspicious lesions by classifying images from total body photography as part of a targeted, nationwide melanoma screening programme in Australia (see Table 3 for scenario). This potential clinical workflow was endorsed by only 25.4%; however, a large minority were unsure (30.3%). When asked if this workflow was acceptable, 41.9% said not at all, or only slightly. However, 32.8% found it moderately acceptable (only 10.6% thought the workflow was highly or completely acceptable).

**TABLE 3:** A potential clinical workflow using AI for melanoma screening.

Clinical workflow scenario: “A patient undergoes total body photography performed by a trained dermatology imaging technician (melanographer) as part of a targeted nationwide melanoma screening program in Australia: Trained technician identifies suspicious lesions and performs total body photography (2D or 3D TBP) and dermoscopic images of all suspicious lesions. Technician also examines areas unseen by the imaging technology (e.g., scalp) and makes additional annotations as needed. AI tool then classifies both macroscopic (from TBP) and dermoscopic images and generates an AI report. AI generated report sent to a virtual dermatologist to review, sign off and create a management plan.”

Item	Response	Results *n* (%)
Would you endorse this clinical workflow?	Yes	31 (25.4%)
	No	36 (29.5%)
	Unsure	37 (30.3%)
	Missing data	18 (14.8%)
How acceptable is this screening workflow to you?	Not at all	23 (18.9%)
	Slightly	28 (23%)
	Moderately	40 (32.8%)
	Highly	12 (9.8%)
	Completely	1 (0.8%)
	Missing data	18 (14.8%)

### 3.5 The Role of the Australasian College of Dermatologists in Preparing for AI in Dermatology

Participants were asked to provide feedback on how the Australasian College of Dermatologists (ACD) should prepare for the deployment of AI in dermatology. A thematic analysis of the 67 open‐ended responses centred on four major ways the ACD should act, as shown in Table 4: (1) Education—provide training and upskilling for dermatologists on AI and how to use AI; (2) Monitoring—set up ways to pro‐actively monitor, investigate and assess AI developments as they are occurring, evaluate the prospects of AI tools, and advise on risks/benefits; (3) Collaboration—collaborate and consult with AI developers in order to facilitate clinician input on the direction of AI development and intended use; (4) Lead and advocate—lead in developing guidelines and policies on AI use, as well as advocate for regulations on AI that hold developers to account.

**TABLE 4:** What should the Australasian College of Dermatologists (ACD) do in preparation for the deployment of AI in dermatology?

Theme	Representative excerpts
(1) Education	“Training, upskilling, offer short sessions at ASM, to state based meetings.” “Train fellows in AI understanding and upskill.” “Have training modules available. College to have on‐line training.”
(2) Monitoring	“Monitor and assess various platforms and advise fellows of findings.” “Be informed of what changes and tools there are in the AI field and which will impact the practice of dermatology in future.” “Taskforce to understand risks and benefits.” “Monitor its development and assess cost benefits.”
(3) Collaboration	“Consultation with companies that own this technology to ensure clinicians can have a key voice in how this technology is used.” “Collaboration with AI providers and the legal profession to come up with a tool that is highly accurate and legal cover that does not expose the dermatologist to negligence claims against the end‐provider.” “Maybe work with tech companies.” “We need to be involved and be a stakeholder in this space.”
(4) Lead and Advocate	“Be at the forefront, drive policy, control the use and direction.” “Look extremely hard at the ethics and evidence and ensure they are not being pushed by medical technology companies to steal money from the public purse.” “Standardised rules/recommendations on appropriate use of AI.” “Work with TGA for appropriate licensing of AI.” “Have a committee to be involved in all legal and medical aspects of AI related to dermatology.” “Advocate for regulations around the use of AI technology.” “Aggressively advise on it.” “Lobby government for legislative change.”

## 4 Conclusions

The narrative in health care has increasingly shifted from AI feasibility towards AI integration [4, 5, 15], and this survey provides the most up to date exploration of attitudes and use of AI among dermatologists in Australia. The results show that use of AI for clinical and administrative purposes appears to have grown since Scheetz et al. [14] conducted their 2021 survey of Australian dermatologists, which likely reflects the availability of AI tools through their incorporation into skin imaging platforms (despite the fact that no AI tool for melanoma diagnosis has been granted TGA approval in Australia) and the popularity of generative AI tools such as ChatGPT. Where Scheetz et al. [14] found that only 14.4% had ever used AI, this has increased considerably in the current survey to 43.4% for clinical purposes and 42.7% for administrative purposes (e.g., writing patient notes). However, only a minority were using AI regularly for clinical (12.3%) or administrative (17%) purposes. Most AI use is relatively infrequent and occurred only rarely. While this could indicate that these clinicians are comfortable using AI and find that they currently only need it in rare circumstances, it may also simply reflect instances of isolated usage borne out of experimental curiosity on the part of clinicians (and perhaps more frequent usage or experimentation is curbed by dissatisfying results, or inadequate AI knowledge). Indeed, our results show that the number of participants who rated the accuracy of currently available AI tools as poor/fair was around five times higher than those who rated AI accuracy as good/excellent, and the proportion of participants who were unwilling to trust AI (39.4%) far exceeded those who were trusting of AI (24.6%).

Nevertheless, the most widely expected benefits of AI endorsed by most participants were its potential to provide more targeted referrals to specialist care and to improve patient access to screening. This may indicate that dermatologists see more value in AI as a triage tool for lesions requiring expert assessment, rather than as a diagnostic aid to experts per se. Indeed, the most widely endorsed potential benefit of AI was that it may reduce the time dermatologists need to spend on monotonous tasks (for some dermatologists, this may include skin checks for low‐risk patients). Notably though, more than half the sample (59.9%) were of the view that their patients are willing for them to use AI for clinical decisions or diagnosis, and this raises the need for co‐designed research to explore how the expectations of consumers and clinicians interact when it comes to AI use.

Currently, the potential benefits of AI anticipated by dermatologists appear to be tempered by several other findings. First, echoing Scheetz et al.'s earlier findings [14], the majority of participants cited AI error as a major concern and most thought that AI tools ought to be at least as accurate as the “best performing” dermatologist in order to be used as a diagnostic aid. This sets a very high bar for intended use that may limit the incorporation of AI into diagnostic decision‐making. Most participants set a lower bar for AI when used as a melanoma screening tool saying that AI need only be at least as accurate as the “average” dermatologist. Nearly all participants said that information about the accuracy and performance indicators of the AI tool was essential/highly important to know to be able to trust AI. In practice the availability of this information from AI may be variable and lacking transparency, particularly with machine learning tools whose quality must be regularly monitored as new data impacts performance [16], therefore the development of labelling standards for AI based software in dermatology should be a high priority [17, 18].

Second, many participants lacked familiarity with the technology and only 24.6% rated their knowledge of AI as good or excellent (similar to the 23.8% found in the 2020 multi‐country survey by Polesie et al. [13]; and slightly less than the 30.5% Ali et al. [8] found among Saudi Arabian dermatologists in 2023). In several areas, this clearly limited the extent to which dermatologists felt they could give confident views about AI. For instance, many participants felt that they did not yet have enough experience to confidently say whether they trusted AI (13.9%) or assess AI's accuracy (41%). Nevertheless, a key finding of this survey is a clear desire expressed by participants for the ACD to provide education for dermatologists about AI, be pro‐active in monitoring AI developments, create clear policies for AI use, inform the direction of AI development and use through engagement with AI researchers and developers, and be a voice for clinicians by advocating for regulations.

This study is the largest survey of dermatologists in Australia to date, although a higher response rate (16.2%) would have improved generalizability of the findings. In context however, most previous surveys exploring dermatologists' attitudes towards AI have either not reported response rates [8, 10, 13], or found a lower response rate than the current study (e.g., Nelson et al. [11] survey with American Academy of Dermatology (AAD) 3.9%; Scheetz et al. [14] with ACD 13.2%). Importantly though, this study helps to address the relative paucity of empirical evidence about clinician attitudes towards AI in dermatology in Australia and provides a useful platform for future investigations. For instance, there is not yet a consensus on where AI tools ought to be positioned in the process—perhaps as an expert guide to decision making, as an adjunct to decision making, or only as a second opinion on difficult cases [4, 19]. Furthermore, it isn't clear whether AI is intended, or likely, to benefit all clinicians (e.g., experts and/or novices), and in which circumstances. Even in other areas of healthcare where AI has become much more integrated into daily use, such as radiology, recent surveys have found that although clinicians have generally positive attitudes towards AI and use it often, many clinicians are still uncertain about the extent to which AI improves decision‐making (see for example radiologists' perceptions [20]). As such, there is an opportunity for studies using more in‐depth qualitative methods to explore how dermatologists wish to employ AI within their decision‐making processes.

Taken together, our findings indicate that although some dermatologists are beginning to use AI tools (including for administrative tasks), there remains wariness and distrust among dermatologists about the accuracy of AI tools for diagnostic purposes. However, most participants thought AI would perform important tasks within dermatology (few thought AI would replace dermatologists outright) and there is clearly interest among dermatologists for AI to play a role in skin lesion screening if sufficiently accurate AI tools can be shown to be of benefit. Detailing clinical workflows that incorporate AI in a way that is acceptable to clinicians will be the challenge for developers, policy makers, and regulators, and this is where knowledge outreach from Dermatology Colleges, such as the ACD, may help clinicians to arrive at well informed views.

## Ethics Statement

Ethics approval for this study was granted by The University of Queensland's Human Ethics Committee (approval number 2024/HE000542).

## Conflicts of Interest

H.P.S. is a shareholder of MoleMap NZ Limited and e‐derm consult GmbH and undertakes regular teledermatological reporting for both companies. H.P.S. is a Medical Consultant for Canfield Scientific Inc. and a Medical Advisor for First Derm.

## Data Availability

The data that support the findings of this study are available from the corresponding author upon reasonable request.
